# Smoking cessation attempts and successes among Nunavimmiut

**DOI:** 10.17269/s41997-023-00790-5

**Published:** 2023-07-06

**Authors:** Yohann Courtemanche, Natalia Poliakova, Gina Muckle, Richard E. Bélanger

**Affiliations:** 1 Centre de Recherche du CHU de Québec-Université Laval, Québec, Québec Canada; 2 École de Psychologie, Université Laval, Québec, Québec Canada; 3 Département de Pédiatrie, Université Laval, Québec, Québec Canada

**Keywords:** Smoking cessation, Inuit, Tobacco, Quebec, Arrêt de la consommation de tabac, Inuit, tabagisme, Québec

## Abstract

**Objectives:**

The smoking rate in Canada has declined in past decades, yet smoking rates remain high in Nunavik (northern Québec), where an estimated 80% of adult respondents smoke. We investigated sociodemographic factors, smoking behaviours, harm perception, and social support as determinants of smoking cessation attempts and successes among Nunavimmiut.

**Methods:**

Past year smoking frequency, quantity smoked, and cessation attempts and aids were documented in a sample of 1326 Nunavimmiut aged 16 and over in the *Qanuilirpitaa?* 2017 survey. Sociodemographic indicators, social support, cessation aids, and smoking harm perception were investigated as potential determinants. All factors were modeled by logistic regressions and adjusted for age and sex.

**Results:**

Thirty-nine percent of smokers tried to quit smoking in the preceding year, and 6% of those were successful. Older Nunavimmiut (aOR = 0.84 [0.78, 0.90]) and those smoking 20 + cigarettes/day (aOR = 0.94 [0.90, 0.98]) were less likely to attempt to quit. Ungava coast residents (aOR = 1.87 [1.36, 2.57]), separated/widowed/divorced individuals (aOR = 2.43 [1.09, 5.38]), and occasional smokers (aOR = 2.77 [1.61, 4.76]) compared to those living on the Hudson coast, single individuals, and daily smokers, respectively, were more likely to report cessation attempts. Most used no particular cessation aid (58%), 28% relied on family/self-help/support programs, and 26% used medication. Women were more likely to rely on spirituality/traditional methods (aOR = 1.92 [1.00, 3.71]) and less likely to rely on electronic cigarettes (aOR = 0.33 [0.13, 0.84]), as were older participants (aOR = 0.67 [0.49, 0.94]). Those with more years of schooling were more likely to rely on electronic cigarettes (aOR = 1.47 [1.06, 2.02]). These estimates are prone to biases due to the relatively low participation rate in the survey (37%).

**Conclusion:**

Despite many attempts reported by participants, regional partners of this study underlined that successful smoking cessation remains a challenge for many Nunavimmiut. Key differences were identified in approaches and determinants of smoking cessation attempts, but most smokers did not use cessation aids. These results are in line with the experience of the Inuit partners of this study and can inform targeted public health interventions to support the many Nunavimmiut trying to quit smoking, notably increasing accessibility and acceptability of cessation aids. Inuit partners of this study highlighted the importance for interventions and communication efforts to reflect Nunavik’s context.

## Résumé

**Objectifs:**

Le taux de tabagisme a diminué au Canada dans les dernières décennies, mais il demeure élevé au Nunavik (Nord du Québec) où on estime que 80 % des participants adultes fument. Cet article étudie les facteurs sociodémographiques, les habitudes tabagiques, le support social et la perception du risque comme déterminants de la cessation tabagique chez les Nunavimmiut.

**Méthode:**

Les habitudes de tabagisme (fréquence, quantité, tentatives d’abandon) sur 12 mois ont été documentées chez 1 326 Nunavimmiut (16 ans et plus) dans l’enquête Qanuilirpitaa? 2017. Tous les facteurs considérés comme déterminants potentiels ont été modélisés par régression logistique (ajustement pour âge et sexe).

**Résultats:**

Trente-neuf pourcent des fumeurs ont tenté d’arrêter et 6 % de ceux-ci ont réussi. Les Nunavimmiut plus âgés (RCa = 0,84[0,78, 0,90]) et ceux fumant 20 + cigarettes/jour (RCa = 0,94[0,90, 0,98]) étaient moins susceptibles d’avoir rapporté une tentative d’abandon. Les résidents de l’Ungava (RCa = 1,87[1,36, 2,57]), ceux séparés/divorcés/veufs (RCa = 2,43[1,09, 5,38]) et les fumeurs occasionnels (RCa = 2,77[1,61, 4,76]), comparés aux résidents de l’Hudson, aux célibataires et aux fumeurs quotidiens, respectivement, étaient plus susceptibles d’avoir tenté un abandon. La majorité n’ont utilisé aucune aide (58 %), 28 % se sont tournés vers la famille/auto-assistance/groupe de soutien et 26 % a eu recours à la médication. Les femmes utilisaient plus fréquemment la spiritualité ou des méthodes traditionnelles (RCa = 1,92[1,00, 3,71]) et moins fréquemment la cigarette électronique (RCa = 0,33[0,13, 0,84]), tout comme les plus âgés (RCa = 0,67[0,49, 0,94]). Ceux ayant complété plus d’années d’éducation utilisaient plus la cigarette électronique (RCa = 1,47[1,06, 2,02]). Ces estimés pourraient être biaisés puisque le taux de participation à l’enquête était relativement faible (37 %).

**Conclusion:**

Malgré plusieurs tentatives rapportées par les participants, les partenaires régionaux de cette étude ont souligné que la cessation tabagique demeure un défi pour plusieurs Nunavimmiut. Différentes approches et des déterminants de tentatives de cessation ont été identifiés, mais la majorité n’ont pas utilisé d’aide à la cessation. Ces résultats, qui cadrent avec l’expérience des partenaires Inuit de cette étude, peuvent guider des interventions ciblées pour supporter les nombreux Nunavimmiut tentant de cesser de fumer, notamment faciliter l’accès et l’acceptabilité des méthodes de cessation. Les partenaires Inuit ont souligné l’importance d’avoir des interventions et méthodes de communication adaptées au contexte du Nunavik.

## Introduction

Tobacco-attributable morbidity and mortality not only affect families and communities, but also generate economic costs and put a strain on healthcare services (World Health Organization, 2019).

While smoking prevalence in Canada has drastically declined from 49.5% in 1965 to 15.1% in 2017, the tobacco smoking (cigarette smoking, hereafter “smoking”) rate in Nunavik remains high, with 80% of the population aged 16 and over being smokers in 2017, 90% of whom smoked daily (Bélanger et al., 2020; Reid et al., 2019). These rates have been observed for decades with little change (Bélanger et al., 2020; Bougie & Kohen, 2017). Of the four regions in Inuit Nunangat, all but Nunavik saw a reduction of daily smoking between 1991 and 2012 (Bougie & Kohen, 2017). Young Nunavimmiut aged 16 to 30 years old are particularly at risk, with between 70.2% and 77.9% of them smoking on a daily basis in 2017 (Bélanger et al., 2020).

In a 2004 populational survey of Nunavimmiut’s health, 42% of daily smokers and 65% of occasional smokers reported having tried to quit smoking during the preceding year (Plaziac et al., 2007). Most of those attempting to quit smoking reported being motivated by the health impacts of smoking (Plaziac et al., 2007), but a majority of them used no specific cessation aids (64% for daily and 79% for occasional smokers). Medication, which includes patches and gum, was used by less than a quarter of those attempting to quit (24%) (Plaziac et al., 2007).

Around the globe, strategies to reduce tobacco use in Indigenous communities have effectively reduced initiation, reduced consumption, and increased quit rates (Minichiello et al., 2016). Smoking cessation products (nicotine replacement therapy (NRT) or other medication) and phone-based behavioural interventions appear to be as effective in non-Indigenous as in Indigenous populations (Hayward et al., 2007; Johnston et al., 2013; Smith et al., 2014). However, the need for culturally adapted resources and interventions remains. For example, while the use of national quit lines by Indigenous smokers in Canada has led to successful cessation attempts, targeted resources should notably take into account the culture and language, housing, and availability of health services (Inuit Tapiriit Kanatami, 2014).

Some interventions in Indigenous communities have shown positive outcomes on reduced initiation and consumption and increased quit rates in empirical studies, but interventions aiming at a change in the community prevalence of tobacco use were inconclusive (Minichiello et al., 2016). These contrasting results also highlight the need for multi-faceted interventions (Minichiello et al., 2016). Furthermore, a review of research on smoking cessation in Indigenous populations of Australia, New Zealand, Canada, and the United States concluded that successful interventions needed to be integrated, flexible, and community-based and to address known barriers and facilitators of cessation (DiGiacomo et al., 2011).

The primary objective of this study is to describe and investigate smoking cessation attempts and successes among Nunavimmiut in the year preceding the Qanuilirpitaa? 2017 survey, and their determinants.

## Methods

### Qanuilirpitaa? 2017 survey

The *Qanuilirpitaa?* 2017 Nunavik Inuit Health Survey was conducted among 1326 Nunavimmiut aged 16 and over in all 14 Nunavik communities. This survey, spearheaded by the Nunavik Regional Board of Health and Social Services (NRBHSS), is a joint initiative of community leaders and regional organizations. The main objective of this survey was to update the health profile of the region since the last large-scale survey conducted in 2004.

A complex multistage sampling plan was used to obtain a non-proportional stratified sample of the entire Nunavik population aged 16 and over. A list of all residents was obtained from the regional government prior to the survey, and was adjusted to take into account recent relocations (within and outside of Nunavik) as well as recent deaths. If a resident identified on the list was not able to participate, efforts were made to find a replacement participant with similar characteristics. In addition to the objective of representativeness for the entire population, providing accurate estimations in subgroups of interest was a main goal of the survey. To achieve this, the Nunavik population was stratified according to sex, age (16–19, 20–30, and 31 + years old) and community (14 communities). Power analyses were conducted to evaluate the number of participants required in each stratum for comparison between groups (*α* = 0.05, *β* = 0.80). The overall response rate was 31% for people aged 16 to 30 years old and 42% for people aged 31 years and over. This relatively low response rate was mainly due to non-contact rates and was taken into consideration in the computation of the sampling weights. Statisticians at the Institut national de santé publique du Québec computed sampling weights taking into account the sample design, non-response at recruitment, non-response on specific instruments, and adjustments to match sociodemographic characteristics of the Nunavik population (post-stratification).

### Community engagement and ethics

The *Qanuilirpitaa?* 2017 Nunavik Inuit Health Survey was set up following a resolution adopted by the NRBHSS requesting that a new health survey be conducted to update the information on the health status of Nunavimmiut. This survey was conducted in partnership with major Nunavik organizations, the Institut national de santé publique du Québec, and researchers from Université Laval, McGill University, and Trent University. An Inuit-led Steering Committee oversaw the preparation, conduct, data interpretation, and dissemination of the survey results in accordance with the First Nations principles of Ownership, Control, Access, and Possession (OCAP®; First Nations Information Governance Centre, 2021). A Data Management Committee (DMC) evaluated the usefulness of the research questions for the region, and approved data and biological sample requests. This committee brings together representatives from the NRBHSS and the health centres, the Kativik Regional Government, Makivik Corporation, Kativik Ilisarniliriniq, Avataq Cultural Institute, and Qarjuit Youth Council. The proposal of this article was submitted to and evaluated by the DMC and community representatives in spring 2020 to ensure that the proposed analyses were within the scope of the survey’s original objectives. Thereafter, the first draft of this manuscript was presented to our Inuit collaborators and the results were discussed and co-interpreted in spring 2021. The final manuscript and a plain language summary were shared with Inuit partners of this study. The *Qanuilirpitaa?* 2017 survey was also approved by the ethics committee of the Centre de recherche du CHU de Québec. Informed written consent was obtained from each participant and a clinical follow-up for abnormal results was undertaken when needed. Detailed information on survey procedures is provided in the Methodological Report (Hamel et al., 2020).

### Measures

All sociodemographic and smoking characteristics were documented by computerized and interviewer-administered questionnaires (available in Inuktitut, English, and French). Current cigarette smoking status was documented as daily, occasional, or not at all. Cessation attempt was documented with the question “In the past 12 months, did you stop smoking for at least 24 h because you were trying to quit? Yes/No.”

The analytical sample was composed of smokers (either daily or occasional at the time of the survey or having quit in the preceding year) in the year preceding the 2017 survey. Successful cessation in the year preceding the survey was based on current smoking status and cessation in the previous year (i.e., smoking at some point in the past year, but no longer at the time of the survey). Cessation aids were documented by a series of Yes/No, non-exclusive statements for: spirituality/traditional methods; family/self-help or support programs; nicotine patches; nicotine gum; pills (Zyban/Champix); e-cigarette/vapor; no method (cold turkey and other unspecified, non-recognized cessation aids/techniques) and other means. For analytic purposes, NRTs (nicotine patches, nicotine gum) and prescription pills (Zyban/Champix) were combined as medication. For spiritual or traditional methods, no formal definition was presented in the questionnaire to allow participants to identify themselves if their cessation attempt included a spiritual or traditional component. The questionnaires were published in the Appendix of the thematic report on substance use in Nunavik (Bélanger et al., 2020).

#### Determinants of smoking cessation

Potential determinants tested for their association were selected a priori based on current literature of factors associated with cessation. In terms of sociodemographic factors, sex, age, marital status, education, income, community size, and coast of residence (Hudson coast is the western and Ungava coast is the eastern coast of Northern Québec) were investigated. Smoking behaviours, such as current smoking status (daily, occasional) and number of cigarettes smoked daily, were also tested. Finally, harm perception of regular tobacco smoking (no to moderate risk vs. great risk) and social support were considered. Social support was measured as a continuous score derived from 5 items documented by questionnaire, a higher score representing a higher level of social support (Richmond, 2009).

### Statistical analyses

Variables were categorized to facilitate the interpretation of multivariate models by using the adjusted odds ratio (aOR) as the measure of association. Sociodemographic and smoking characteristics were compared between men and women by Chi-square test. Multivariate associations, adjusting for sex and age, were tested by logistic regressions. SAS 9.4 (SAS Institute Inc., Cary, NC) software was used for all analyses. We adopted a twofold approach in our exploratory investigation of factors associated with smoking cessation. First, sociodemographic and smoking behaviours were put in relation with cessation attempts and successes in the previous year among all past-year smokers (*n*_unweighted_ = 1043). Second, we tested the association between these factors and the choice of smoking cessation aids among past-year smokers who tried to quit (*n*_unweighted_ = 404). Given the exploratory nature of our investigation and the limited number of respondents in some categories, we tested factors individually with adjustment for age and sex.

Sampling weights and bootstrap replicate weights were used in all computations to obtain statistically unbiased parameter and variance estimates. Detailed weighting procedures are available in the survey’s methodological report (Hamel et al., 2020). Chi-square test and logistic regression were used to test all associations.

## Results

### Descriptive statistics of the 2017 sample

Descriptive characteristics of smokers, who represent 79.5% of a sample of Nunavimmiut weighted to be representative of the entire Nunavik population, are presented in Table 1. The vast majority of smokers smoked on a daily basis (90.0%), but the majority smoked less than 20 cigarettes per day (79.7%). The majority of smokers considered regular tobacco smoking to pose great risk of harm (56.5%). Two out of five smokers stopped smoking for at least 24 h because they were trying to quit in the year preceding the survey (39.3%). Of those, 6.1% successfully quit, which represents 2.4% of all smokers. Most reported using no specific method (58.4%), while family, self-help, or support programs (28.2%) and medication (25.8%) were both used by about a quarter of smokers. Spirituality or traditional methods (12.9%) and the electronic cigarette (7.1%) were less frequently used as cessation aids.

**Table 1:** Descriptive statistics of Nunavimmiut smokers in 2017 (*n*_unweighted_* = *1043)

Variable	Proportion or mean (95% CI)			*p-*value comparing sexes^3^
	Total	Women	Men	
Sex	-	50.77	43.23	
Age (years)				
16–30	46.70 (45.17, 48.23)	48.16 (46.60, 49.72)	45.19 (42.57, 47.81)	0.15
31–54	40.12 (38.18, 42.05)	38.95 (36.62, 41.27)	41.33 (38.26, 44.39)	
55 +	13.18 (11.49, 14.88)	12.90 (10.84, 14.95)	13.48 (10.74, 16.21)	
Marital status				
Single	45.19 (41.67, 48.70)	45.12 (41.24, 49.00)	45.25 (39.51, 51.00)	0.14
Married or common law	50.03 (46.42, 53.65)	48.59 (44.55, 52.63)	51.52 (45.77, 57.28)	
Separated, divorced, or widowed	4.78 (3.41, 6.15)	6.29 (4.36, 8.21)	3.23 (1.27, 5.18)	
Education (years of schooling; mean)	9.16 (9.02, 9.30)	9.25 (9.08, 9.43)	8.96 (8.71, 9.20)	0.06
Employment (working)	67.64 (64.41, 70.87)	66.27 (62.69, 69.86)	69.05 (63.83, 74.27)	0.38
Income				
Under $20,000 per year	44.08 (40.39, 47.76)	41.72 (37.44, 45.99)	46.24 (40.22, 52.26)	0.24
$20,000 or more per year	55.92 (52.24, 59.61)	58.28 (54.01, 62.56)	53.76 (47.74, 59.78)	
Community size				
Large community	58.23 (56.53, 59.93)	59.00 (57.38, 60.63)	57.44 (54.46, 60.43)	0.51
Small community	41.77 (40.07, 43.47)	41.00 (39.37, 42.62)	42.56 (39.57, 45.54)	
Coast of residence				
Hudson coast	59.32 (57.87, 60.77)	58.85 (57.33, 60.37)	59.81 (57.36, 62.26)	0.51
Ungava coast	40.68 (39.23, 42.13)	41.15 (39.63, 42.67)	40.19 (37.74, 42.64)	
Social support score (mean)	13.30 (13.07, 13.53)	13.90 (13.62, 14.20)	12.50 (12.11, 12.91)	< 0.001
Smoking status				
Daily	90.03 (88.01, 92.05)	90.82 (88.56, 93.09)	89.21 (85.68, 92.74)	0.45
Occasional	9.97 (7.95, 11.99)	9.18 (6.91, 11.44)	10.79 (7.26, 14.32)	
Number of cigarettes smoked^1^				
Less than 20	79.72 (76.77, 82.67)	85.45 (82.27, 88.63)	73.76 (68.48, 79.03)	0.0002
20 or more	20.28 (17.33, 23.23)	14.55 (11.38, 17.73)	26.24 (20.97, 31.52)	
Harm perception of regular smoking				
No risk to moderate risk	43.54 (40.32, 46.77)	40.20 (36.56, 43.83)	46.99 (41.42, 52.56)	0.27
Great risk	56.46 (53.23, 59.68)	59.80 (56.17, 63.44)	53.01 (47.44, 58.58)	
Cessation attempt in past year (% yes)^1^	39.28 (36.07, 42.50)	36.85 (33.19, 40.51)	41.78 (36.23, 47.33)	0.15
Cessation method used (non-exclusive)^2^				
No specific method	58.40 (52.99, 63.82)	57.99 (52.01, 63.98)	58.78 (50.16,67.40)	0.88
Family, self-help, or support program	28.18 (23.54, 32.82)	27.84 (22.15, 33.53)	28.48 (21.19, 35.78)	0.89
Medication	25.83 (20.99, 30.68)	25.85 (20.19, 31.50)	25.82 (18.24, 33.41)	1.00
Spirituality or traditional methods	12.87 (9.25, 16.49)	9.14 (5.58, 12.71)	16.24 (9.99, 22.49)	0.04
Electronic cigarette	7.09 (4.10, 10.09)	3.78 (1.55, 6.01)	10.09 (4.84, 15.33)	0.01
Successful cessation in past year (% yes)^1^	2.38 (1.40, 3.35)	2.41 (1.24, 3.58)	2.34 (0.70, 3.98)	0.95

^1^Among smokers

^2^ Among smokers who attempted to quit

^3^ Wald Log-Linear Chi-square test for proportions and *F* tests for means

#### Cessation attempts and successes in the year preceding the 2017 survey

Associations between sociodemographic characteristics, smoking behaviours, and cessation attempts and successes in the year preceding the 2017 survey are presented in Table 2. Older smokers were less likely to attempt to quit smoking than younger smokers (aOR = 0.84 [0.78, 0.90]). Separated, divorced or widowed participants were more likely to attempt to quit smoking than singles (aOR = 2.43 [1.09, 5.38]), as were residents of the Ungava coast compared to the Hudson coast (aOR = 1.87 [1.36, 2.57]). In terms of smoking behaviours, occasional smokers were more likely to attempt to quit smoking than daily smokers (aOR = 2.77 [1.61, 4.76]). Participants smoking a higher number of cigarettes (more than 20 daily) were less likely to attempt to quit (aOR = 0.94 [0.90, 0.98]). None of the sociodemographic or smoking behaviours tested were associated with successful cessation in the year preceding the 2017 survey.

**Table 2:** Determinants of past year smoking cessation attempts and successes among Nunavimmiut smokers in 2017 (*n*_unweighted_ = 1043)

Determinants	OR^1^ (95% CI)	
	Cessation attempt in past year	Successful cessation in past year
Model 1: minimal adjustment		
Sex		
Men	Ref	Ref
Women	0.80 (0.59, 1.09)	0.95 (0.70, 1.28)
Age	0.84 (0.78, 0.90)	1.18 (0.43, 3.23)
Model 2^2^: marital status		
Single	Ref	Ref
Married or common law	1.11 (0.82, 1.51)	1.53 (0.55, 4.24)
Separated, divorced, or widowed	2.43 (1.09, 5.38)	0.85 (0.15, 4.88)
Model 3^2^: education		
Years of schooling	1.02 (0.95, 1.10)	1.04 (0.86, 1.26)
Model 4^2^: employment		
Not working	Ref	Ref
Working	1.18 (0.87, 1.61)	0.70 (0.25, 1.94)
Model 5^2^: income		
Under $20,000 per year	Ref	Ref
$20,000 or more per year	0.92 (0.66, 1.29)	0.36 (0.12, 1.08)
Model 6^2^: community size		
Large community	Ref	Ref
Small community	1.13 (0.84,1.52)	0.54 (0.19, 1.56)
Model 7^2^: coast of residence		
Hudson coast	Ref	Ref
Ungava coast	1.87 (1.36, 2.57)	0.98 (0.37, 2.57)
Model 8^2^: smoking status		
Daily	Ref	-
Occasional	2.77 (1.61, 4.76)	-
Model 9^2^: number of cigarettes smoked		
Less than 20	Ref	-
20 or more	0.94 (0.90, 0.98)	-
Model 10^2^: harm perception regular		
No risk to moderate risk	Ref	Ref
Great risk	0.95 (0.70, 1.28)	1.89 (0.62, 5.73)
Model 11^2^: social support		
Continuous 5-item score	1.01 (0.97,1.05)	1.06 (0.96, 1.17)

^1^Odds ratios (attempt yes vs. no; successful cessation yes vs. no) obtained by logistic regression with sampling weights

^2^Models 2 to 11 adjusted for sex and age

### Cessation aids used in the year preceding the 2017 survey

Among the factors investigated, only sex, age, education, and social support were related to the type of smoking cessation aid used by Nunavimmiut (Table 3). Women were more likely than men to rely on spirituality or traditional methods to help them quit smoking (aOR = 1.92 [1.00, 3.71]). On the other hand, women were less likely than men to have used the electronic cigarette as a cessation aid (aOR = 0.33 [0.13, 0.84]), as were older participants compared to younger ones (aOR = 0.67 [0.49, 0.94]). Higher education level was positively associated with the use of electronic cigarettes as a cessation aid (aOR = 1.47 [1.06, 2.02]). Higher social support was positively associated with relying on family, self-help, or support programs to quit smoking (aOR = 1.14 [1.06, 1.22]).

**Table 3:** Determinants of cessation aid use among Nunavimmiut smokers attempting to quit in 2017 (*n*_unweighted_ = 404)

Determinants	No method used	Family, self-help, or support programs	Spirituality or traditional methods	Medication	Electronic cigarette
	OR (95% CI)	OR (95% CI)	OR (95% CI)	OR (95% CI)	OR (95% CI)
Model 1: minimal adjustment					
Sex: women vs. men	0.97 (0.63, 1.50)	0.95 (0.59, 1.52)	1.92 (1.00, 3.71)	1.02 (0.62, 1.69)	0.33 (0.13, 0.84)
Age	1.01 (0.91, 1.12)	0.89 (0.78, 1.01)	1.01 (0.87, 1.18)	1.11 (0.99, 1.23)	0.67 (0.49, 0.94)
Model 2: marital status (ref: single)					
Married or common law, separated, divorced, or widowed	0.80 (0.52, 1.24)	0.98 (0.61, 1.55)	1.26 (0.57, 2.78)	0.91 (0.59, 1.39)	0.33 (0.05, 2.03)
Model 3: education					
Years of schooling	1.10 (0.98, 1.24)	0.97 (0.85, 1.10)	1.02 (0.86, 1.22)	1.04 (0.93, 1.18)	1.47 (1.06, 2.02)
Model 4: employment					
Working vs. not	1.00 (0.63, 1.59)	0.84 (0.51, 1.38)	0.89 (0.44, 1.78)	0.95 (0.54, 1.66)	1.35 (0.22, 8.52)
Model 5: income					
$20,000 or more per year vs. less than $20,000 per year	0.89 (0.53, 1.49)	1.10 (0.62, 1.94)	1.03 (0.50, 2.16)	0.63 (0.36, 1.09)	0.81 (0.25, 2.63)
Model 6: community size					
Small community vs. large	0.75 (0.47, 1.19)	0.67 (0.40, 1.12)	1.18 (0.60, 2.33)	1.42 (0.85, 2.36)	0.43 (0.07, 2.64)
Model 7: coast of residence (ref = Hudson)					
Ungava coast	1.26 (0.81, 1.97)	0.87 (0.52, 1.45)	1.07 (0.54, 2.12)	0.83 (0.48, 1.41)	1.12 (0.41, 3.05)
Model 8: smoking status					
Occasional vs. daily	1.61 (0.81, 3.18)	0.94 (0.44, 1.98)	0.64 (0.19, 2.15)	0.55 (0.24, 1.26)	0.64 (0.01, 84.23)
Model 9: number of cigarettes smoked					
20 or more vs. less than 20	0.98 (0.91, 1.05)	1.01 (0.97, 1.06)	1.03 (0.95, 1.06)	1.02 (0.96, 1.10)	1.02 (0.95, 1.09)
Model 10: harm perception regular					
Great risk vs. no to moderate risk	1.46 (0.91, 2.33)	0.69 (0.40, 1.17)	0.48 (0.22, 1.03)	1.38 (0.80, 2.39)	8.25 (0.01, 999)
Model 11: social support					
Continuous 5-item score	0.94 (0.88, 1.00)	1.14 (1.06, 1.22)	1.03 (0.93, 1.14)	1.00 (0.94, 1.08)	1.14 (0.94, 1.40)

Odds ratios (cessation aid used yes vs. no) obtained by logistic regression with sampling weights. Models 2 to 11 adjusted for sex and age

## Discussion

Smoking cessation was and remains a challenge in Nunavik, with many Nunavimmiut taking steps to quit smoking. The high rates of tobacco smoking and of cessation attempts, as well as the low success rate for cessation, were expected by Inuit partners of this study. The low success rate of cessation is not specific to Nunavik, as any smokers may go through multiple attempts before successfully quitting (Chaiton et al., 2016). Yet, the rate seen in the present analysis is quite low compared to rates reported in other populations. Taking into account major differences with the Inuit population and cautions in comparing such results, a representative survey of American adults reported that 13% of those attempting successfully quit, compared to 7% in this study (Babb et al., 2017). Age, marital status, region of residence, and smoking behaviours (number of cigarettes smoked daily and occasional use) were associated with past-year cessation attempts. These factors are not specific to Nunavimmiut and have also been identified as determinants in Western, non-Indigenous populations (Osler & Prescott, 1998). Higher social support was associated with a more frequent use of family, self-help, or support programs for smoking cessation. This may indicate that these individuals may be more confident to quit with those close to them, but conversely that those with lower social support may need special support from health professionals.

Some challenges are however specific to Nunavik communities, notably the social environment and access to resources. The latter may explain regional differences in smoking cessation attempts and the higher odds for Ungava coast, where Kuujjuaq, the largest village and seat of the regional government, is located. As a result of the high smoking rate, Nunavimmiut trying to quit or who recently quit are likely to be in frequent contact or living with smokers, a known risk factor associated with higher tobacco use and lower quit rates (Bougie & Kohen, 2018; Osler & Prescott, 1998; Smith et al., 2014). A change in the family structure, and thus spousal influence, may explain the increased odds of smoking cessation among separated, divorced, or widowed participants (Homish & Leonard, 2005).

Most smokers were aware that smoking poses great risk to their health, yet a higher perception of harm was not associated with cessation attempts or successes. In other populations, risk perception and health literacy have been associated with smoking cessation (Jacobson et al., 2014; Stewart et al., 2013). Evidence-based and accessible information on the risk of tobacco smoking are still essential to public health interventions, as many smokers may not accurately understand the risk (Krosnick et al., 2017).

While health literacy may increase contemplation or attempts of smoking cessation, adequate access to services is central to successful cessations: in the current literature, the success rate of unassisted attempts is 50% lower than with any kind of assistance (Zhu et al., 2000). While one-off events—for example the “Quit to win challenge”—have been popular in Nunavik, resources are needed to follow up with those trying to quit and help them deal with day-to-day challenges. In discussions with local representatives, they identified the need for more long-term follow-up in the communities to better support smokers trying to quit. While we found no significant association between social support and cessation, other studies in Indigenous populations have highlighted the need for both individual and group-based support as a key tool to increase smoking cessation (Lancaster & Stead, 2017; Stead et al., 2017).

Achieving long-term smoking cessation requires sustained efforts and specifically dedicated resources to support those trying to quit in every community. Local representatives highlighted the need for these programs to be tailored to Nunavimmiut’s reality. An example given by Inuit partners of this study was the graphic health warnings printed on all tobacco packaging in Canada that feature non-Inuit individuals and messages in French or English, which may not speak to Nunavik smokers. To address this, public health professionals have already developed stickers to be added to the packaging that feature Inuit stories with warnings written in Inuktitut. When carefully implemented, culturally adapted interventions have the potential to increase engagement and effectiveness (Minichiello et al., 2016; Nierkens et al., 2013).

Medication was used by only a quarter of those who tried to quit, despite being available free of charge in all communities. Currently, Nunavimmiut smokers need to put in a request with their healthcare provider to obtain these medications, which could limit access if they are not aware of this treatment option or not comfortable asking for it. Medications have the potential to be a significant tool in reducing smoking rates, as all forms of NRT have been shown to help people trying to quit, with increased rates between 50% and 70% (Stead et al., 2012). These first-line therapies, as well as other treatment options such as prescription pills, should be considered with and presented as success enhancers to the patient and a shared decision should be made to better suit individual preferences.

The high rate of smoking among youth in Nunavik is cause for concern: 70.2% of those aged 16–20 and 77.9% of those aged 21–30 reported smoking on a daily basis (Bélanger et al., 2020). While younger Nunavimmiut were more likely to attempt to quit smoking, they were not any more likely to be successful. Of note, youth were more likely to rely on electronic cigarettes or vaping products as a cessation aid. Electronic cigarettes are a relatively new product that was not designed to be used as a cessation aid, and studies on its effectiveness, compared to NRT or no treatment, are promising but remain uncertain (Hartmann-Boyce et al., 2020). On the contrary, use of electronic cigarettes during adolescence has been shown to increase the risk to initiate smoking subsequently (Baenziger et al., 2020). As a cessation aid or not, electronic cigarettes are most popular among the new generation of Nunavimmiut (Bélanger et al., 2020) and further studies are needed to investigate the relationship between electronic cigarettes, cigarette smoking, and smoking initiation and cessation in Nunavik.

To our knowledge, this study is the first large-scale investigation of smoking cessation to take place in Nunavik. While this study mainly relies on a quantitative assessment of smoking cessation and related factors, the input of Inuit partners represents a major strength of this study. The research objectives were formulated with local and public health representatives to align with the needs of the community. The results were co-interpreted with community leaders to provide a culturally informed interpretation that adequately represents their experiences in the communities. Many sociodemographic characteristics and smoking behaviours were taken into account, but other important factors were not considered: notably, living conditions (house crowding, food security) and traumatic events (residential schooling) that have been associated with smoking in Inuit Nunangat (Bougie & Kohen, 2018). The investigation of these factors may further our understanding of smoking and smoking cessation and guide public health interventions. The sampling design covered the Nunavik population aged 16 and over, but 78.3% of current and former smokers smoked their first cigarette before the age of 16 and half started smoking daily before that age (Bélanger et al., 2020). Therefore, this study does not cover all smokers in Nunavik and the results may not be fully applicable to very young and new smokers. Participation bias may also be present if individuals with certain characteristics—notably relating to smoking behaviours—refused or were unable to participate, which could potentially bias our estimates of smoking rates and smoking cessation. Furthermore, the participation rate in the survey was relatively low: 31% for individuals aged 16–30 years and 42% for those aged 30 years and older (Hamel et al., 2020). Given the information collected in this survey, it is not possible to evaluate for how long respondents had quit smoking, only that it occurred in the year preceding the survey. While less precise than the CDC’s measure (minimum of 6 months cessation required (Creamer et al., 2019)), this measure of smoking cessation is similar to the “short-term quitters” definition of the Canadian Tobacco and Nicotine Survey (Statistics Canada, 2020). The low prevalence of successful cessations restricted the use of multivariate models to include potential confounders and limited statistical power to test potential determinants. Also, some categories with low frequencies (such as some modalities of marital status) had to be combined for some models. Residual confounding, notably by age, may contribute to some of the associations reported, for example the higher odds of cessation attempts among separated, divorced, or widowed smokers. Finally, as with any self-reported data, our measures of smoking behaviours may be influenced by social desirability bias and thus potentially biasing our estimates.

## Conclusion

This study identified key factors associated with smoking cessation attempts and the cessation aids used. Given the low rate of long-term cessation in this sample, these determinants can guide public health interventions to reduce smoking. Further research should focus primarily on long-term successful cessation to increase the power to identify determinants and effective cessation aids. Since most smokers did not use cessation aids, increasing all efforts to support those trying to quit is key. Notably, increasing accessibility and acceptability towards cessation aids is paramount. Inuit partners of this study highlighted that to meet the needs of smokers trying to quit, interventions need to be adapted to Nunavik’s context in terms of the type of interventions and the communication to Nunavimmiut. Reducing the smoking rate in Nunavik has the potential to drastically improve the health and quality of life of individuals and communities for generations to come.

## Contributions to knowledge

What does this study add to existing knowledge?

- Despite many cessation attempts, smoking rates remain high and stable in Nunavik.
- Key differences according to sex, age, education, and region of residence were identified in approaches and determinants of cessation attempts.

What are the key implications for public health interventions, practice, or policy?

- Smokers trying to quit need support to achieve successful cessation.
- Given the variety of smoking cessation aids used, targeted approaches may better suit the needs of smokers trying to quit in Nunavik.

## Data Availability

Inquiries regarding access to the data should be addressed to the survey’s Data Management Committee.

## References

[CR1] Babb S, Malarcher A, Schauer G, Asam K, Jamal A (2017). Quitting smoking among adults — United States, 2000–2015. MMWR. Morbidity and Mortality Weekly Report.

[CR2] Baenziger, O. N., Ford, L., Yazidjoglou, A., Joshy, G., & Banks, E. (2020). E-cigarette use and combustible tobacco cigarette smoking uptake among non-smokers, including relapse in former smokers: Umbrella review, systematic review and meta-analysis. *MedRxiv*. 10.1101/2020.09.16.20195438 10.1136/bmjopen-2020-045603 PMC8011717 33785493

[CR3] Bélanger, R. E., Muckle, G., Courtemanche, Y., & Poliakova, N. (2020). *Substances use. Nunavik Inuit Health Survey 2017 Qanuilipitaa? How are we now?* Nunavik Regional Board of Health and Social Services (NRBHSS) & Institut national de santé publique du Québec (INSPQ).

[CR4] Bougie E, Kohen DE (2017). Smoking prevalence among Inuit in Canada. Health Reports. Statistics Canada..

[CR5] Bougie E, Kohen DE (2018). Smoking correlates among Inuit men and women in Inuit Nunangat. Health Reports. Statistics Canada..

[CR6] Chaiton M, Diemert L, Cohen JE, Bondy SJ, Selby P, Philipneri A, Schwartz R (2016). Estimating the number of quit attempts it takes to quit smoking successfully in a longitudinal cohort of smokers. British Medical Journal Open.

[CR7] Creamer, M. R., Wang, T. W., Babb, S., Cullen, K. A., Day, H., Willis, G., Jamal, A., & Neff, L.. (2019). Tobacco product use and cessation indicators among adults — United States, 2018*.**Morbidity and Mortality Weekly, 68*(45), 1013–1019. 10.15585/mmwr.mm6845a2 10.15585/mmwr.mm6845a2 PMC6855510 31725711

[CR8] DiGiacomo M, Davidson PM, Abbott PA, Davison J, Moore L, Thompson SC (2011). Smoking cessation in indigenous populations of Australia, New Zealand, Canada, and the United States: Elements of effective interventions. International Journal of Environmental Research and Public Health.

[CR9] First Nations Information Governance Centre. (2021). *The First Nations Principles of OCAP*®. Resource website. https://fnigc.ca/ocap-training/. Accessed 4 Jan 2023.

[CR10] Hamel, D., Hamel, G., & Gagnon, S. (2020). *Methodological report*. Nunavik Inuit Health Survey 2017 Qanuilirpitaa? How are we now? Quebec: Nunavik Regional Board of Health and Social Services (NRBHSS) & Institut national de santé publique du Québec (INSPQ). http://www.nrbhss.ca/sites/default/files/health_surveys/A11991_RESI_Rapport_methodologique_EP4.pdf. Accessed 4 Jan 2023.

[CR11] Hartmann-Boyce, J., McRobbie, H., Lindson, N., Bullen, C., Begh, R., Theodoulou, A., Notley, C., Rigotti, N. A., Turner, T., Butler, A. R., Fanshawe, T. R., & Hajek, P. (2020). Electronic cigarettes for smoking cessation. *Cochrane Database of Systematic Reviews*, *10*. 10.1002/14651858.CD010216.pub4 10.1002/14651858.CD010216.pub4 PMC8094228 33052602

[CR12] Hayward LM, Campbell HS, Sutherland-Brown C (2007). Aboriginal users of Canadian quitlines: An exploratory analysis. Tobacco Control.

[CR13] Homish GG, Leonard KE (2005). Spousal influence on smoking behaviors in a US community sample of newly married couples. Social Science & Medicine.

[CR14] Inuit Tapiriit Kanatami. (2014). *Social determinants of Inuit health in Canada*. Ottawa, ON: Inuit Tapirit Kanatami. https://www.itk.ca/wpcontent/uploads/2016/07/ITK_Social_Determinants_Report.pdf. Accessed 4 Jan 2023.

[CR15] Jacobson JD, Catley D, Lee HS, Harrar SW, Harris KJ (2014). Health risk perceptions predict smoking-related outcomes in Greek college students. Psychology of Addictive Behaviors : Journal of the Society of Psychologists in Addictive Behaviors.

[CR16] Johnston V, Westphal DW, Glover M, Thomas DP, Segan C, Walker N (2013). Reducing smoking among indigenous populations: New evidence from a review of trials. Nicotine & Tobacco Research : Official Journal of the Society for Research on Nicotine and Tobacco.

[CR17] Krosnick JA, Malhotra N, Mo CH, Bruera EF, Chang L, Pasek J, Thomas RK (2017). Perceptions of health risks of cigarette smoking: A new measure reveals widespread misunderstanding. PLoS ONE.

[CR18] Lancaster T, Stead LF (2017). Individual behavioural counselling for smoking cessation. The Cochrane Database of Systematic Reviews.

[CR19] Minichiello A, Lefkowitz ARF, Firestone M, Smylie JK, Schwartz R (2016). Effective strategies to reduce commercial tobacco use in Indigenous communities globally: A systematic review. BMC Public Health.

[CR20] Nierkens V, Hartman MA, Nicolaou M, Vissenberg C, Beune EJAJ, Hosper K, van Valkengoed IG, Stronks K (2013). Effectiveness of cultural adaptations of interventions aimed at smoking cessation, diet, and/or physical activity in ethnic minorities. A systematic review. PLOS ONE.

[CR21] Osler M, Prescott E (1998). Psychosocial, behavioural, and health determinants of successful smoking cessation: a longitudinal study of Danish adults. Tobacco Control.

[CR22] Plaziac, C., Hamel, D., & Rochette, L. (2007). *Qanuippitaa? Tobacco use*. https://numerique.banq.qc.ca/patrimoine/details/52327/46572. Accessed 26 Jun 2023.

[CR23] Reid, J., Hammond, D., Tariq, U., Burkhalter, R., Rynard, V., & Douglas, O. (2019). *Tobacco Use in Canada: Patterns and trends, 2019 Edition*. www.tobaccoreport.ca. Accessed 4 Jan 2023.

[CR24] Richmond CAM (2009). The social determinants of Inuit health: A focus on social support in the Canadian Arctic. International Journal of Circumpolar Health.

[CR25] Smith SS, Rouse LM, Caskey M, Fossum J, Strickland R, Culhane JK, Waukau J (2014). Culturally-tailored smoking cessation for Adult American Indian smokers: A clinical trial. The Counseling Psychologist.

[CR26] Statistics Canada. (2020). *Canadian Tobacco and Nicotine Survey (CTNS): Summary of results for 2019*. https://www.canada.ca/en/health-canada/services/canadian-tobacco-nicotine-survey/2019-summary.html#n4. Accessed 4 Jan 2023.

[CR27] Stead LF, Perera R, Bullen C, Mant D, Hartmann-Boyce J, Cahill K, Lancaster T (2012). Nicotine replacement therapy for smoking cessation. The Cochrane Database of Systematic Reviews.

[CR28] Stead LF, Carroll AJ, Lancaster T (2017). Group behaviour therapy programmes for smoking cessation. The Cochrane Database of Systematic Reviews.

[CR29] Stewart DW, Adams CE, Cano MA, Correa-Fernández V, Li Y, Waters AJ, Wetter DW, Vidrine JI (2013). Associations between health literacy and established predictors of smoking cessation. American Journal of Public Health.

[CR30] World Health Organization. (2019). *Tobacco Fact Sheet*. https://www.who.int/en/news-room/fact-sheets/detail/tobacco. Accessed 4 Jan 2023.

[CR31] Zhu S, Melcer T, Sun J, Rosbrook B, Pierce JP (2000). Smoking cessation with and without assistance: A population-based analysis. American Journal of Preventive Medicine.

